# Histology, physiology, and transcriptomic and metabolomic profiling reveal the developmental dynamics of annual shoots in tree peonies (*Paeonia suffruticosa* Andr.)

**DOI:** 10.1093/hr/uhad152

**Published:** 2023-08-01

**Authors:** Ningning Tong, Qingyan Shu, Baichen Wang, Liping Peng, Zheng'an Liu

**Affiliations:** State Key Laboratory of Plant Diversity and Specialty Crops, Institute of Botany, Chinese Academy of Sciences, Beijing 100093, China; China National Botanical Garden, Beijing 100093, China; University of Chinese Academy of Sciences, Beijing 100049, China; State Key Laboratory of Plant Diversity and Specialty Crops, Institute of Botany, Chinese Academy of Sciences, Beijing 100093, China; China National Botanical Garden, Beijing 100093, China; China National Botanical Garden, Beijing 100093, China; University of Chinese Academy of Sciences, Beijing 100049, China; Photosynthesis Research Center, Key Laboratory of Photobiology, Institute of Botany, Chinese Academy of Sciences, Beijing 100093, China; State Key Laboratory of Plant Diversity and Specialty Crops, Institute of Botany, Chinese Academy of Sciences, Beijing 100093, China; China National Botanical Garden, Beijing 100093, China; State Key Laboratory of Plant Diversity and Specialty Crops, Institute of Botany, Chinese Academy of Sciences, Beijing 100093, China; China National Botanical Garden, Beijing 100093, China

## Abstract

The development of tree peony annual shoots is characterized by “withering”, which is related to whether there are bud points in the leaf axillaries of annual shoots. However, the mechanism of “withering” in tree peony is still unclear. In this study, *Paeonia ostii* ‘Fengdan’ and *P. suffruticosa* ‘Luoyanghong’ were used to investigate dynamic changes of annual shoots through anatomy, physiology, transcriptome, and metabolome. The results demonstrated that the developmental dynamics of annual shoots of the two cultivars were comparable. The withering degree of *P. suffruticosa* ‘Luoyanghong’ was higher than that of *P. ostii* ‘Fengdan’, and their upper internodes of annual flowering shoots had a lower degree of lignin deposition, cellulose, C/N ratio, showing no obvious sclerenchyma, than the bottom ones and the whole internodes of vegetative shoot, which resulted in the “withering” of upper internodes. A total of 36 phytohormone metabolites were detected, of which 33 and 31 were detected in *P. ostii* ‘Fengdan’ and *P. suffruticosa* ‘Luoyanghong’, respectively. In addition, 302 and 240 differentially expressed genes related to lignin biosynthesis, carbon and nitrogen metabolism, plant hormone signal transduction, and zeatin biosynthesis were screened from the two cultivars. Furtherly, 36 structural genes and 40 transcription factors associated with the development of annual shoots were highly co-expressed, and eight hub genes involved in this developmental process were identified. Consequently, this study explained the developmental dynamic on the varied annual shoots through multi-omics, providing a theoretical foundation for germplasm innovation and the mechanized harvesting of tree peony annual shoots.

## Introduction

The tree peony (*Paeonia suffruticosa* Andr.) is a perennial woody shrub and a unique plant resource native to China, belonging to the family Paeoniaceae [1]. Its flowers, roots, and seeds have ornamental, medicinal, and edible values, respectively [2]. In addition, the shoots and leaves of tree peony can be used as special feed additives for animal husbandry. The plant of this species grows slowly, and the actual annual growth of the tree peony is only 1/3 to 1/4 of its annual shoot growth. The reason for this is because there exists “withering” phenomenon in the annual shoots of tree peony [3]. This phenomenon is similar with the self-pruning, also known as self-cutting of apical bud, which has been observed in many woody plants, such as *Gossypium* spp., *Vitis vinifera*, and *Citrus sinensis*. Previous studies indicated that self-pruning was a developmental boundary of all kinds of shoots and had relationship with flower differentiation. The self-pruning was involved in a process of abscission of plant organs that the tip of the branches shed or died from the plant body [4–6]. However, there is still some different between “withering” in tree peony and self-pruning. In tree peony, there are usually 8 to 10 leaf axillaries in the annual shoots, only three to four leaf axillaries with buds at the bottom of annual flowering shoots can be lignified, while the upper parts without axillary buds cannot be lignified, then leading withering and abscission; but if an apical bud is formed, the annual shoots will not wither, all lignified [3]. The phenomenon of withered shoots in tree peony is extremely unique, but the process of abscission also observed in the “withering”. In the process of abscission of plant organs, it is usually observed that genes that affect the development of abscission zone, the balance of plant hormone, and the content of carbohydrates play an important role in this process [4]. Although many researches focus on the process of abscission, but the molecular mechanism of “withering” in tree peony is still unclear.

In the “withering” process of tree peony, the reason for the abscission of the upper part of annual flowering shoots is its degree of lignification lower than that in the bottom with buds and varies in different cultivars. Lignification is a stage of plant development caused by lignin deposition on the cell wall [7, 8, 9]. Lignin mainly provides mechanical hardness and strength for cell wall, and promotes the formation of xylem vessels [10]. Lignin is a characteristic component of secondary cell wall, and its precursors are produced by phenylpropane metabolic pathway which regulated by *PAL*, *C4H*, and *4CL* related genes. Previous studies indicated that the mutation of four *PAL* alleles in *Arabidopsis thaliana* resulted in a significant decrease in lignin content, and similar results were found in inhibition of *C4H* gene expression [11, 12]. In addition, *C3H*, *COMT*, *CCoAOMT*, and *F5H*-related genes participate in the specific synthesis pathway of upstream gene regulation of lignin, and changes in these genes not only affect the proportion of various monomers in lignin but also may affect the total amount of lignin [13–15]. Furthermore, *CCR* and *CAD*-related genes also participate in the downstream gene regulation of lignin monomer specific synthesis pathway [16]. Lignin biosynthesis and its molecular mechanism of transcriptional regulation have always been a difficult and hot topic in the research, and to understand the lignification process is important for revealing the “withering” phenomenon in tree peony.

Lignin biosynthesis is closely related to plant hormone signaling pathways. Almost all plant endogenous hormones play a role in the abscission of plant organs, and they can affect plant development at extremely low concentrations [17]. Auxin regulates plant growth and development mainly through the expression of auxin response protein Aux/IAA, auxin response factor ARF, and SAUR and GH3 gene families. They play a role in regulating root development, cell expansion, organ abscission, and leaf senescence [18–21]. Auxin signal function depends on the interaction of ARF-Aux/IAA. The overexpression of *PtoARF5.1* and *PtoIAA9m* inhibited lignin deposition and secondary xylem development [22]. The variety and content of gibberellin vary greatly with the developmental stages and parts of plants, and its main function is to promote cell expansion, tissue differentiation, internode elongation, and other processes [23, 24]. The DELLA protein in *Arabidopsis*, as a negative regulator, plays an inhibitory role in gibberellin signal transduction [25]. Gibberellin promoted xylem development and lignin deposition in the root of sweet potato and inhibited the yield of storage roots by regulating the expression of lignin biosynthesis genes [26]. Cytokinin participates in shoot tip growth, lateral root and bud formation, developmental transition, and leaf senescence [27–29]. The ideal plant-type rice was formed by the regulation of tiller number by auxin and cytokinin [30]. Abscisic acid is a growth inhibitor based on isoprene, which regulates germination, organ abscission, and tissue senescence [31–33]. Plant hormone interactions regulate plant life processes. For example, the interaction between auxin and cytokinin includes synergism and antagonism, which negatively regulate the production of lignin and antagonize each other during plant morphogenesis. In addition, cytokinin promotes auxin transport, which triggers the degradation or glycosylation of cytokinin [34]. Gibberellin and auxin synergistically regulate plant internode elongation. Auxin promotes gibberellin accumulation, and gibberellin triggers auxin transport [35]. Abscisic acid prevents root prolongation by inhibiting cytokinin signal transduction [31].

The transportation and distribution of carbon and nitrogen in different developmental parts of shoots may affect their degree of lignification. Carbon and nitrogen constitute many important compounds and affect plant morphogenesis and organogenesis [36, 37]. Carbon and nitrogen metabolism regulate nutrient supply at different stages of plant development [38]. The dynamic changes between them can affect the formation of photosynthates, protein synthesis, and mineral nutrient absorption [39]. Maintaining the synergy of carbon and nitrogen metabolism and the ratio balance of carbon and nitrogen metabolites (C/N) in cells are crucial for plant development and yield formation [40]. During plant senescence, the transport of nutrients to young tissues can regulate the C/N balance [41]. The C/N balance regulated the growth of *Arabidopsis* seedlings, the expression of photosynthetic genes, and the reactivation of storage lipids, rather than just carbon or nitrogen acting [42]. On the contrary, C/N imbalance can trigger organ senescence [43, 44].

The “withering” characteristic of tree peony annual shoots is an interesting developmental phenomenon that may be regulated by various factors. However, the developmental dynamics of tree peony annual shoots and their developmental mechanisms during the annual growth cycle are not yet clear. To reveal the developmental dynamics of tree peony annual shoots, nine key developmental stages of the two tree peony cultivars were selected to investigate the lignification process and lignin accumulation rules of annual shoots from cytology, and to analyse changes in the secondary cell wall components, as well as carbon and nitrogen from the physiological and biochemical aspects. Furthermore, changes in gene expression and the phytohormone metabolite content in different developmental parts of annual shoots were studied through transcriptome and metabolome analysis, and candidate genes related to the development of annual shoots were screened. This study revealed the developmental dynamics and withering characteristics of tree peony annual shoots, providing a theoretical foundation for germplasm selection and cultivation, the scale production and mechanized harvesting of tree peonies, and contributing to China’s national strategy of ensuring grain and oil security.

## Results

### Dynamic changes in phenotypic characteristics of annual shoots

**Figure 1:**
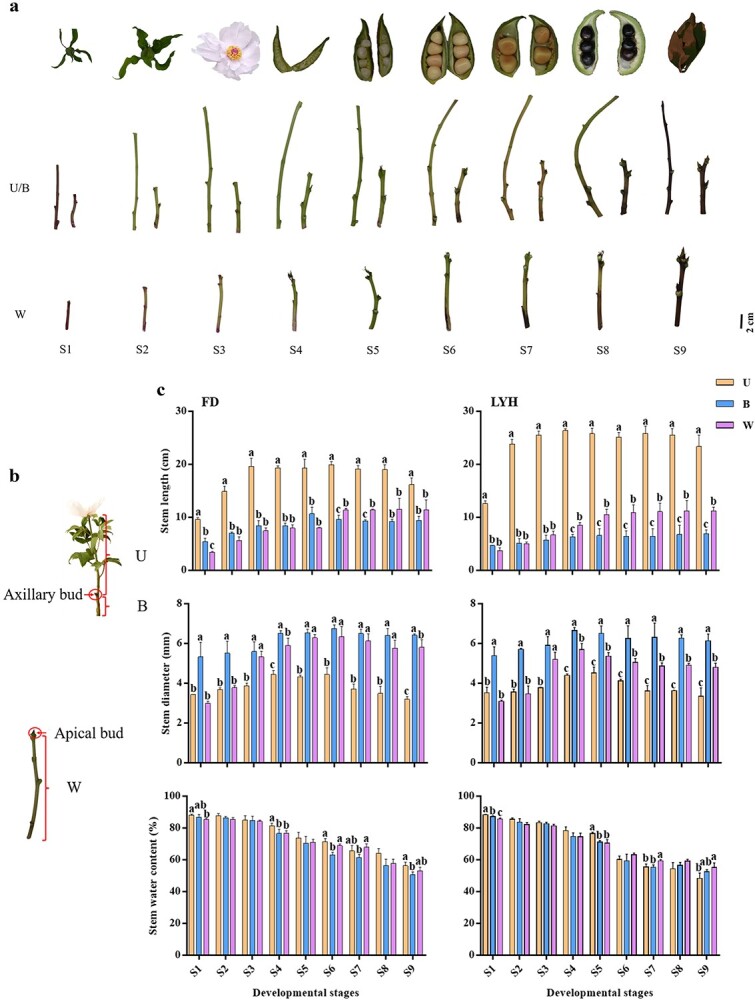
Images and phenotypic characteristics of tree peony annual shoots at nine developmental stages. **a** Images of tree peony annual shoots at nine developmental stages. **b** Images of tree peony annual shoots at three developmental parts. **c** Phenotypic characteristics. FD, *P*. *ostii* ‘Fengdan’; LYH, *P. suffruticosa* ‘Luoyanghong’; U, upper internodes of flowering shoot; B, bottom internodes of flowering shoot; W, whole internodes of vegetative shoot; S1–S9, nine developmental stages. Data are represented as mean ± SD (*n* = 6 stems from the different individual plant), and different letters above error bars indicate significant differences (*p* < 0.05).

*Paeonia ostii* ‘Fengdan’ (FD) and *P. suffruticosa* ‘Luoyanghong’ (LYH), the most widely used oil and ornamental cultivars in China, respectively, were selected for this study. Nine developmental stages were identified during the annual growing season based on the phenological period of tree peony growth, which were extractive branch (S1), unfolding leaves (S2), flowering (S3), 20 to 80 days after flowering (S4-S7), seed maturation (S8), and withering (S9) (Fig. 1a). The collected stems were further divided into the whole (W) internodes of vegetative shoot, as well as the upper (U) and bottom (B) internodes of flowering shoot according to the absence or presence of axillary buds, respectively, based on the developmental characteristics of tree peony annual shoots (Fig. 1b). The phenotypic characteristics of annual shoots were measured (Fig. 1c). The stem length of the U part of the two cultivars first increased, then remained stable, and finally decreased by wilting due to water loss, while B and W did not decrease. The stem length increased sharply from S1 to S3, and primary growth mainly occurred during this stage. The stem length of the U part of LYH was significantly higher than that of FD, while B was lower, and W of both were roughly the same, which indicated the withering degree of LYH was higher than that of FD. In terms of stem diameter, the two cultivars significantly increased at S4, when secondary growth was mainly observed. At almost every stage, B was the thickest, followed by W and U. Moreover, the stem water content of each part of the two cultivars significantly decreased with the developmental stage.

### Dynamic changes in the lignification process of annual shoots

**Figure 2:**
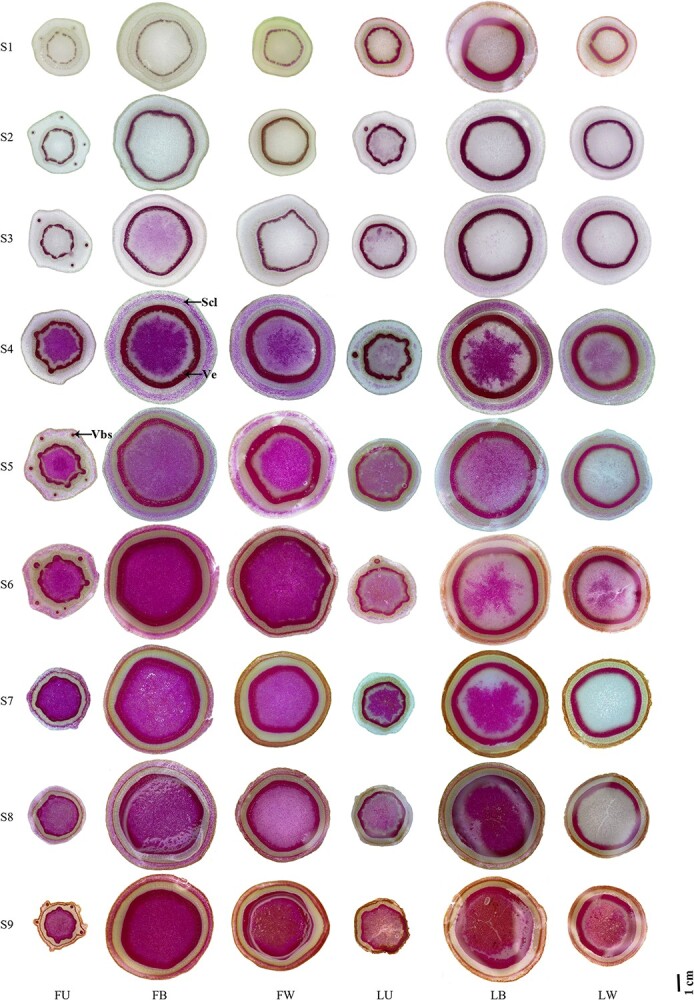
Microscopic observation of tree peony annual shoots at nine developmental stages. FU/FB/FW, upper internodes of flowering shoot/bottom internodes of flowering shoot/whole internodes of vegetative shoot in *P*. *ostii* ‘Fengdan’; LU/LB/LW, upper internodes of flowering shoot/bottom internodes of flowering shoot/whole internodes of vegetative shoot in *P. suffruticosa* ‘Luoyanghong’; S1-S9, nine developmental stages. Details are as follows: Vbs, vascular bundle sheath; Ve, vessel; Scl, sclerenchyma. Bars = 1 cm.

The lignified cell wall (cinnamaldehyde residue in lignin) can be dyed purple red with phloroglucinol staining solution, and its color intensity is consistent with the lignin content. The cell wall color of all parts was very light, only a small amount of lignin was deposited at S1, and there was no significant difference between U, B and W. Lignin deposition accelerated from S2 to S4 in vascular bundle sheaths and vessels, and deposited in sclerenchyma during S4 to S9 (Fig. 2). Compared with U, B and W showed higher lignin deposition, lignin content, and significant sclerenchyma, which could interpret the differences in the degree of lignification leading U withered at low temperatures, while B and W grew normally.

**Figure 3:**
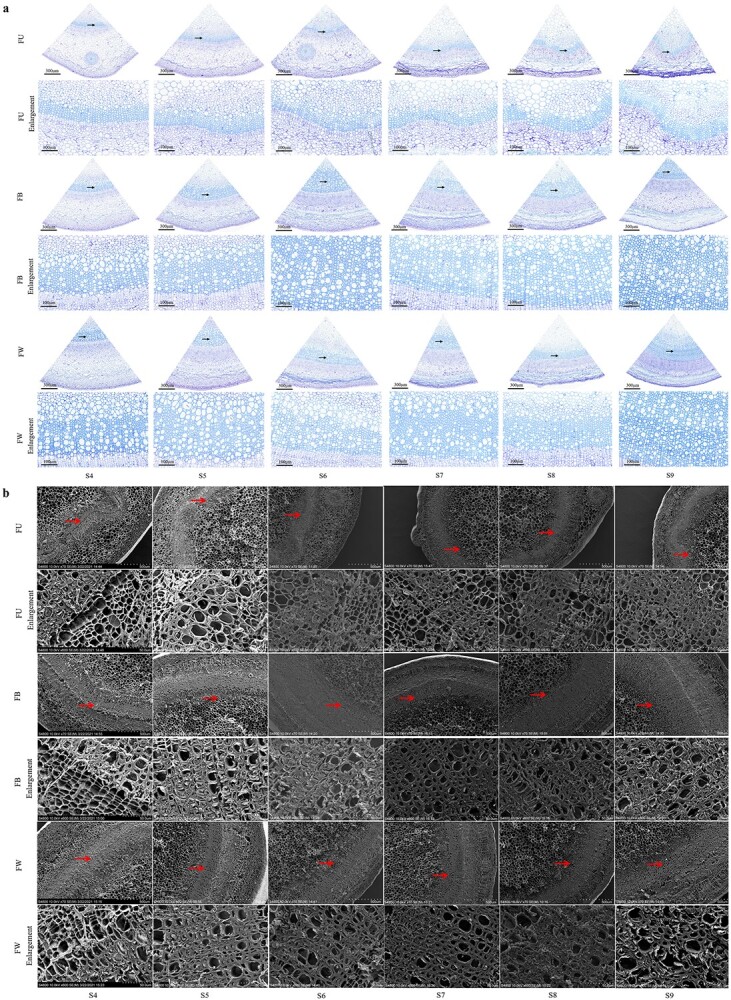
Anatomical analysis and SEM observation of tree peony annual shoots at developmental stages 4–9. **a** Anatomic analysis. The micrographs of partial enlargement of regions are marked by black arrows. FU/FB/FW, bars = 300 μm; Enlargement, bars = 100 μm. **b** SEM observation. The micrographs of partial enlargement of regions are marked by red arrows. FU/FB/FW, bars = 500 μm; Enlargement, bars = 50 μm. FU/FB/FW, upper internodes of flowering shoot/bottom internodes of flowering shoot/whole internodes of vegetative shoot in *P*. *ostii* ‘Fengdan’; S4, 20 days after flowering (DAF); S5, 40 DAF; S6, 60 DAF; S7, 80 DAF; S8, maturity stage; S9, withering stage.

The anatomical morphology of the cell wall and the vascular structure of annual shoots were further observed (Figs. 3a, S1). By the microscopic observation of tree peony annual shoots at nine developmental stages, we could see FD and LYH showed similar lignification process, namely, the elongation growth of annual shoots was completed after S3, and B and W were mainly lignified into woody stems, while U could not complete the lignification process and was unable to overwinter normally, the same fate as herbaceous stems. The number of xylem cells was more in B and W than that in U, which increased gradually with the development of annual shoots. Obvious sclerenchyma appeared in the cortex of B and W at S6. The above results were consistent with the morphological observation of the freehand sections, which together explained the change law of the lignification process of tree peony annual shoots.

### Dynamic changes of secondary cell wall and its components of annual shoots

**Figure 4:**
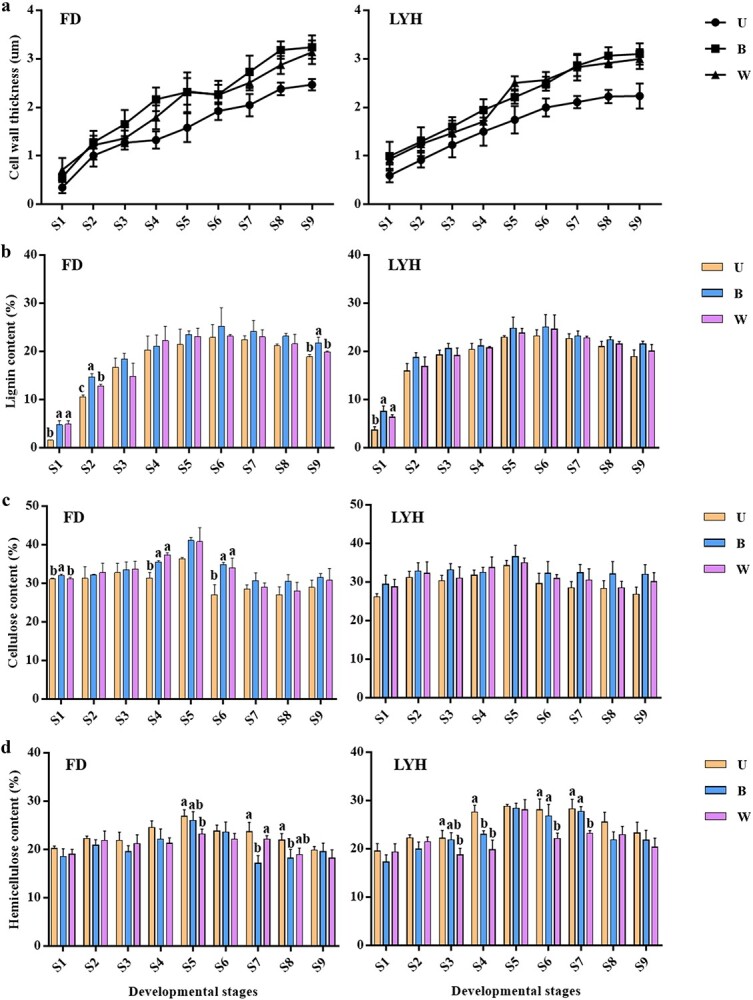
Dynamic changes of cell wall thickness of xylem cells and secondary cell wall composition of tree peony annual shoots at nine developmental stages. **a** The cell walls thickness of xylem cells. **b** Lignin content. **c** Cellulose content. **d** Hemicellulose content. FD, *P*. *ostii* ‘Fengdan’; LYH, *P. suffruticosa* ‘Luoyanghong’; U, upper internodes of flowering shoot; B, bottom internodes of flowering shoot; W, whole internodes of vegetative shoot; S1-S9, nine developmental stages. Data are represented as mean ± SD (*n* = 3), and different letters above error bars indicate significant differences (*p* < 0.05).

As the annual shoots developed, the secondary cell wall grew thicker (Figs. 3b, S2), and the cell wall thickness of xylem cells gradually increased, with B and W thicker than U (Fig. 4a). The total lignin content was determined to verify the results of the above morphological structure (Fig. 4b). The accumulation of lignin content increased continuously from S1 to S6, accelerated from S1 to S2, and slowly decreased from S6 to S9. At the same developmental stage, B had the highest lignin content, followed by W and U, except at S3. The dynamic changes in lignin content and the accumulation of lignin in different developing parts were consistent with the observed results of morphology and structure. In addition, the variation trend of cellulose and hemicellulose content was similar to that of lignin, but was highest at S5. At the same developmental stage, the cellulose content of B and W was higher than that of U, while the hemicellulose content was the opposite (Fig. 4c-d). Therefore, hemicellulose content was abundant in U, while cellulose and lignin content were enrichment in B and W. The difference in the lignin, cellulose, and hemicellulose content in different parts resulted in different lignification degrees, which could be one of the reasons for the withering of tree peony annual shoots.

### Dynamic changes in the carbon and nitrogen content of annual shoots

**Figure 5:**
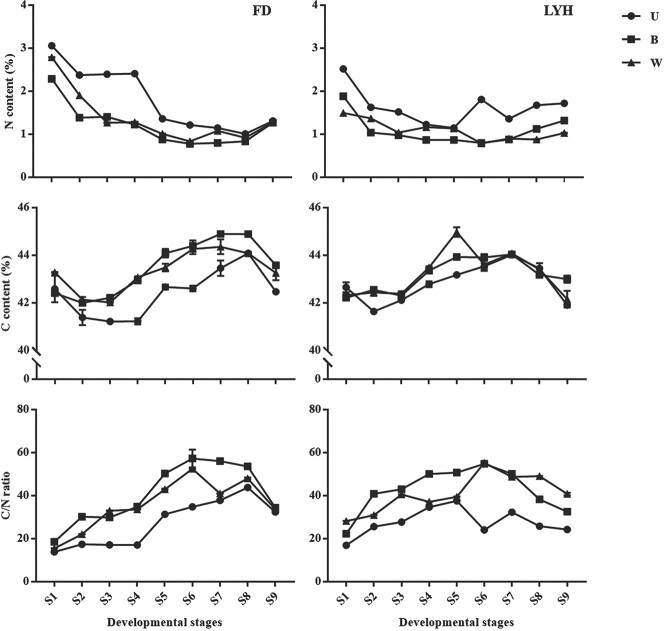
Dynamic changes of C content, N content, and C/N ratio of tree peony annual shoots at nine developmental stages. FD, *P*. *ostii* ‘Fengdan’; LYH, *P. suffruticosa* ‘Luoyanghong’; U, upper internodes of flowering shoot; B, bottom internodes of flowering shoot; W, whole internodes of vegetative shoot; S1-S9, nine developmental stages. Data are represented as mean ± SD (*n* = 3).

The content and ratio of carbon and nitrogen in different developmental stages and parts were analysed to investigate the role of carbon and nitrogen transportation in the growth of tree peony annual shoots (Fig. 5). The results demonstrated that the nitrogen content of annual shoots of the two cultivars decreased as the developmental stage progressed, while the carbon content showed an S-shaped trend. In each developmental stage, the nitrogen content of U was higher than that of B and W, while the carbon content showed the opposite result. In addition, the C/N ratio of B and W was consistently higher than that of U, which may be due to the extremely energy-consuming process during flowering and fruiting of tree peony, and the carbon source of U was transported to the reproductive organs for its flowering and fruiting.

### Analysis of phytohormone metabolites in tree peony annual shoots

**Figure 6:**
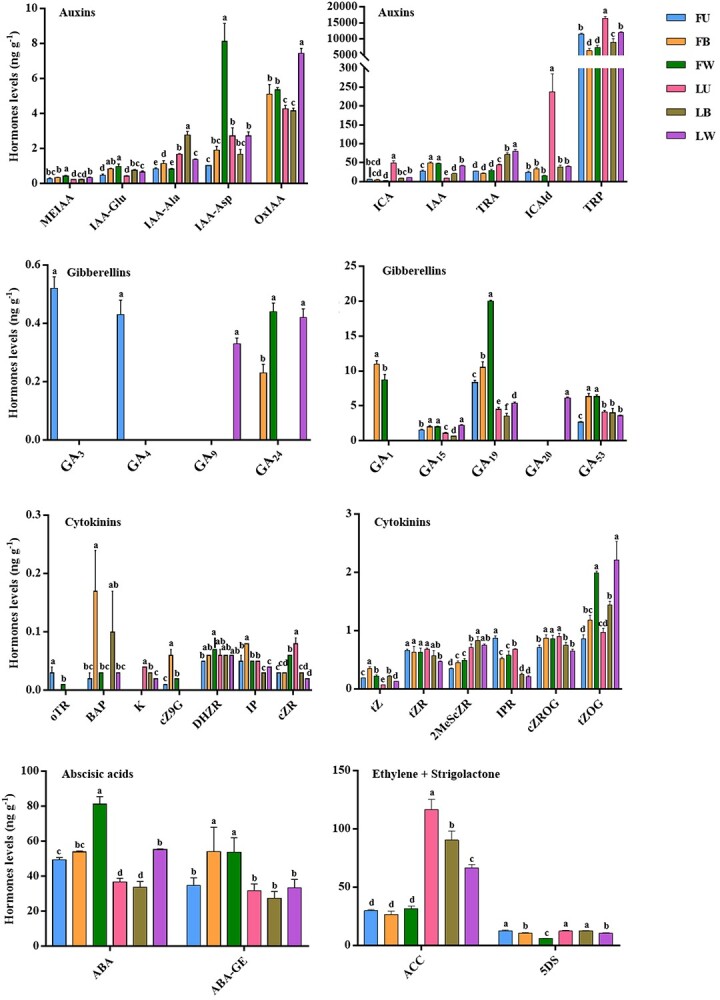
Distribution of phytohormone metabolites in different developmental parts of tree peony annual shoots. FU/FB/FW, upper internodes of flowering shoot/bottom internodes of flowering shoot/whole internodes of vegetative shoot in *P*. *ostii* ‘Fengdan’; LU/LB/LW, upper internodes of flowering shoot/bottom internodes of flowering shoot/whole internodes of vegetative shoot in *P. suffruticosa* ‘Luoyanghong’. Data are represented as mean ± SD (*n* = 3), and different letters above error bars indicate significant differences (*p* < 0.05).

The contents of auxin, gibberellin, cytokinin, abscisic acid, ethylene, and strigolactone in different parts were determined to explore the role of phytohormones in the lignification of tree peony annual shoots. A total of 36 phytohormone metabolites were detected in annual shoots, including 10 auxins, 9 gibberellins, 13 cytokinins, 2 abscisic acids, 1 ethylene, and 1 strigolactone. Their contents are shown in Fig. 6. The results demonstrated that GA_1_ (gibberellin A_1_), GA_3_, GA_4_, cZ9G (cis-zeatin-9-glucoside), and oTR (ortho-topolin riboside) were only detected in FD, while GA_9_, GA_20_, and K (kinetin) were only detected in LYH. The contents of auxin compounds [indole-3-acetic acid (IAA), indole-3-acetyl-glutamic acid (IAA-Glu)], gibberellin compounds (GA_1_, GA_24_), and cytokinin compounds [6-benzyladenine (BAP), trans-zeatin (tZ), trans-zeatin-*O*-glucoside (tZOG), cZ9G] were significantly higher in B and W, while auxin compounds [indole-3-carboxylic acid (ICA), L-tryptophan (TRP)], gibberellin compounds (GA_3_, GA_4_), and cytokinin compounds [IPR (N6-isopentenyladenosine), oTR] in U were more abundant. These phytohormone metabolites accumulated in different developmental parts might regulate the lignification of tree peony annual shoots.

Kyoto encyclopedia of genes and genomes (KEGG) enrichment analysis demonstrated that most of differential metabolites were concentrated in plant hormone signal transduction, metabolic pathways, tryptophan metabolism, biosynthesis of secondary metabolites, diterpenoid biosynthesis, and zeatin biosynthesis pathways (Fig. S3a).

### Full-length transcriptome sequencing and functional annotation of transcripts

PacBio and Illumina sequencing were performed to ascertain the molecular mechanism of tree peony annual shoot development. PacBio cDNA libraries were constructed from mixed plant tissues of FD and LYH during the annual growth season. 68947 and 66929 full-length isoforms of the two cultivars were ultimately obtained for subsequent analysis, respectively (Table S1). Most full-length isoforms (FD: 54439, 78.96%; LYH: 47402, 70.82%) had similar sequences in NR database, and the full-length isoforms (FD: 54867, 79.58%; LYH: 47788, 71.40%) were annotated in at least one database. Additionally, FD and LYH were predicted to have 4316 and 3449 transcription factors (TFs), respectively (Fig. S4).

### RNA-seq and differential expression analysis based on full-length transcriptomes

The full-length isoforms were used as a reference for sequence alignment and Illumina sequencing analysis. An average of 36082275 clean reads were obtained, accounting for 88.14% of total mapping and 44.57% of uniquely mapped isoforms (Table S2). The analysis showed that the sequencing results had high repeatability, which verified that RNA-seq data was reliable (Fig. S5a-d). By pairwise comparison, differentially expressed genes (DEGs) were identified between different developmental parts. The results showed that FW_vs_FU had the highest number of DEGs (4942), followed by LW_vs_LU (4306), while LB_vs_LW had the lowest number of DEGs (748) (Table S3). This indicated that the difference between W and B was small and the difference between W and U was large, which was consistent with the results of plant hormone differential metabolites.

The k-means clustering analysis was used to study the expression patterns of genes in different developmental parts, which divided all DEGs into six clusters (Fig. S6a-b). DEGs in each cluster had similar expression patterns in different developmental parts, suggesting that they could participate in the same pathway or have similar functions. DEGs in cluster 1 of FD and cluster 4 of LYH were primarily expressed at B and W, while the expression patterns of DEGs in cluster 5 of FD and cluster 1 of LYH were opposite, and were mainly expressed at U. These DEGs could be involved in the development and withering of tree peony annual shoots.

The distribution of DEGs obtained by gene ontology (GO) enrichment analysis could clarify the difference in gene function between different developmental parts. The significant enrichment analysis results in GO terms are shown in Fig. S7a-c. Most DEGs were participated in oxidation–reduction and cell wall-related processes. KEGG enrichment analysis indicated that phenylpropanoid biosynthesis, carbon metabolism, nitrogen metabolism, fatty acid metabolism, starch and sucrose metabolism, and photosynthesis were the main pathways of DEGs (Fig. S3b). It can be concluded that the accumulation of bioactive substances and the metabolism of matter and energy were the main processes in the development of tree peony annual shoots.

### Identification and analysis of differentially expressed genes and transcription factors

**Figure 7:**
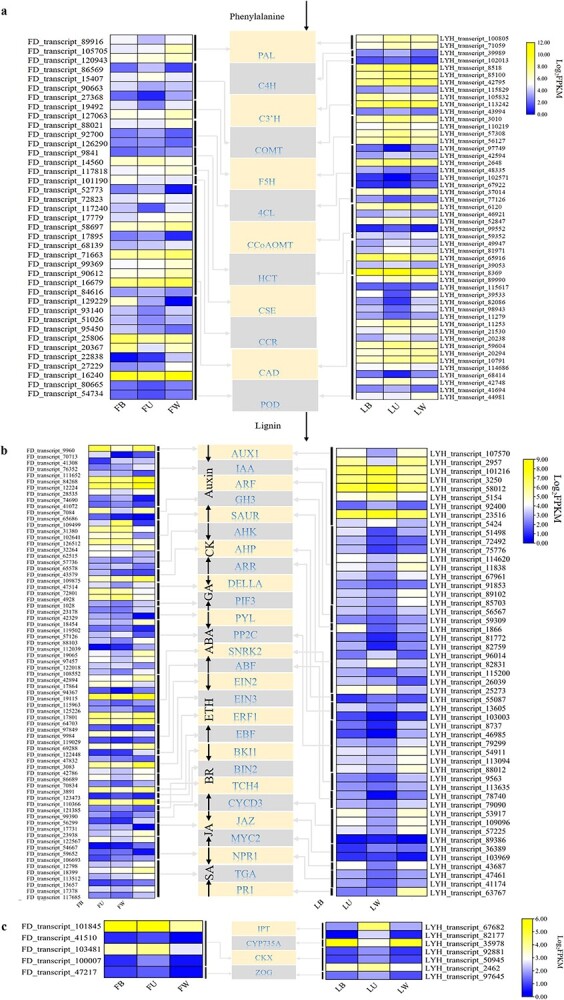
Identification and analysis of DEGs in the three developmental parts of annual shoots of two tree peony cultivars. **a** Expression analysis of DEGs related to lignin biosynthesis pathway. **b** Expression analysis of DEGs related to plant hormone signal transduction pathway. **c** Expression analysis of DEGs related to zeatin biosynthesis pathway. FU/FB/FW, upper internodes of flowering shoot/bottom internodes of flowering shoot/whole internodes of vegetative shoot in *P*. *ostii* ‘Fengdan’; LU/LB/LW, upper internodes of flowering shoot/bottom internodes of flowering shoot/whole internodes of vegetative shoot in *P. suffruticosa* ‘Luoyanghong’.

The three developmental parts of annual shoots of two tree peony cultivars were pairwise compared, and FD and LYH were screened as 39 and 50 DEGs related to lignin biosynthesis, 167 and 112 DEGs related to carbon metabolism, 18 and 20 DEGs related to nitrogen metabolism, 73 and 51 DEGs related to plant hormone signal transduction, 5 and 7 DEGs related to zeatin biosynthesis with a significant difference, respectively, and their expression levels are visualized in Fig. 7 and Fig. S8. The expression levels of caffeic acid 3-*O*-methyltransferase (*COMT*, 2 in FD, 1 in LYH), 4-coumarate-CoA ligase (*4CL*, 1, 3), peroxidase (*POD*, 6, 7) related to lignin biosynthesis, phosphoenolpyruvate carboxykinase (*PCK*, 6, 2), pyruvate kinase (*PK*, 5, 2), 6-phosphogluconolactonase (*PGLS*, 2, 1), 3-hydroxyisobutyryl-CoA hydrolase (*HIBCH*, 1, 1), alcohol dehydrogenase (*ADH*, 5, 4) related to carbon metabolism, and ABA-responsive element binding factor (*ABF*, 3, 2), sucrose non-fermenting 1-related protein kinase 2 (*SNRK2*, 6, 2), protein phosphatase 2C (*PP2C*, 5, 6), cyclin D3 (*CYCD3*, 2, 3) related to plant hormone signal transduction in B and W were higher than those in U, which was consistent with the change rule of lignin content, carbon accumulation, and ABA content, respectively. Conversely, the expression levels of shikimate/quinate hydroxycinnamoyl transferase (*HCT*, 1, 4), cinnamyl alcohol dehydrogenase (*CAD*, 4, 4), *COMT* (2, 6), *4CL* (2, 2) related to lignin biosynthesis, and glyceraldehyde-3-phosphate dehydrogenase (*GAPA*, 1, 7), ribulose-bisphosphate carboxylase small chain (*RBCS*, 5, 5), fructose-bisphosphate aldolase (*ALDO*, 6, 5), fructose-1,6-bisphosphatase (*FBP*, 1, 8), ribose 5-phosphate isomerase A (*RPIA*, 2, 1), isocitrate dehydrogenase (*IDH*, 3, 1), succinyl-CoA synthetase beta subunit (*SUCL*, 1, 1), alanine-glyoxylate transaminase (*AGXT*, 1, 5), phosphoglycolate phosphatase (*PGP*, 1, 1), (S)-2-hydroxy-acid oxidase (*HAO*, 3, 8), glyoxylate/hydroxypyruvate reductase (*HPR*, 1, 2) related to carbon metabolism in U were higher than those in B and W, which was contrary to the accumulation rule of lignin content, and carbon content, respectively. Besides, the expression levels of carbonic anhydrase (*CAN*, 5, 8), cyanate lyase (*CYN*, 1, 1) related to nitrogen metabolism, and cytokinin oxidase (*CKX*, 3, 3), cis-zeatin *O*-glucosyltransferase (*ZOG*, 1, 1) related to zeatin biosynthesis in U were higher than those in B and W, which was consistent with the accumulation rule of nitrogen content, and cytokinin metabolites such as IPR and oTR, respectively. Conversely, the expression level of the nitrate/nitrite transporter (*NRT*, 1, 1) related to nitrogen metabolism was higher in B and W than in U, which was contrary to the accumulation of nitrogen content.

**Figure 8:**
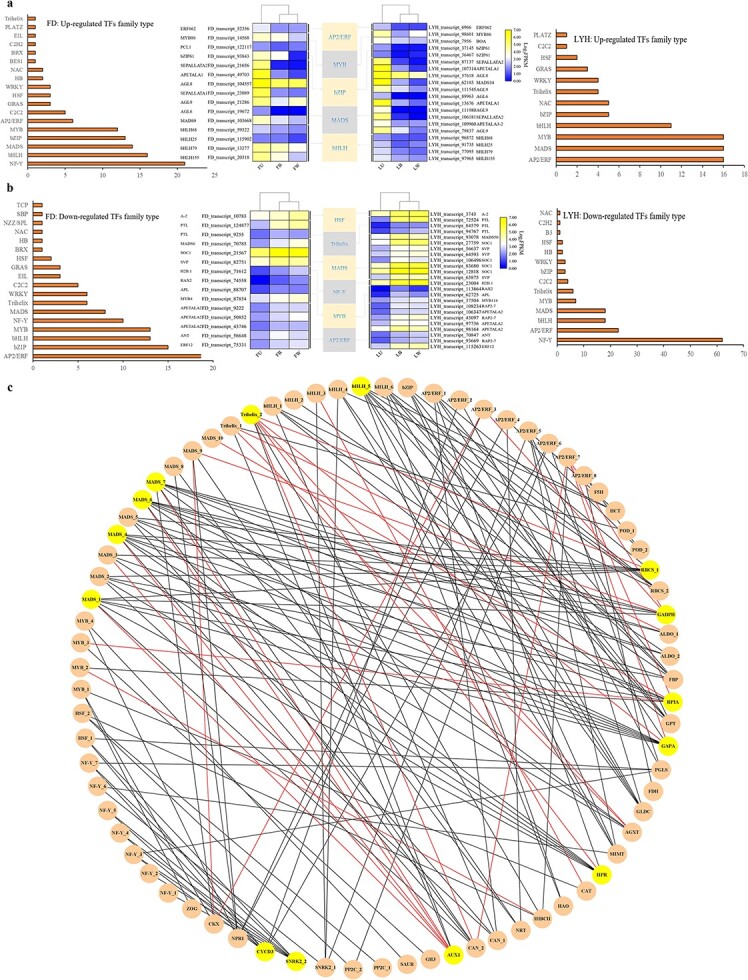
Identification of differentially expressed TFs and co-expression network analysis with candidate structural genes. **a** Identification and analysis of upregulated TFs. **b** Identification and analysis of downregulated TFs. **c** Co-expression network between candidate structural genes and TFs. The black and red lines connecting nodes represent positive and negative correlations, respectively. The thickness of lines stands for Pearson's coefficient. FD, *P*. *ostii* ‘Fengdan’; LYH, *P. suffruticosa* ‘Luoyanghong’.

Twenty-two TF families important for the development of tree peony annual shoots were screened. Among the up-regulated TF family members, the homologous TF families of the two cultivars were MADS, bHLH, MYB, bZIP, and AP2/ERF. They were mainly expressed in U, which was consistent with the accumulation pattern of nitrogen, ICA, TRP, GA_3_, GA_4_, IPR, and oTR (Fig. 8a). Among the down-regulated TF family members, AP2/ERF, MYB, MADS, Trihelix, NF-Y, and HSF were mainly expressed in B and W, which were consistent with the accumulation pattern of lignin, carbon, IAA, IAA-Glu, GA_1_, GA_24_, BAP, tZ, tZOG, and cZ9G (Fig. 8b).

### Co-expression network analysis between candidate structural genes and TFs

The co-expression network of candidate structural genes and TFs associated with the development of annual shoots of the two cultivars was constructed to screen the hub genes (Fig. 8c). The correlation coefficient between genes was calculated using expression data. The correlation threshold was set to 0.9 for a positive correlation and −0.9 for a negative correlation (*p* < 0.05), and the network was visualized using Cytoscape 3.8.0. Nodes with yellow backgrounds indicated stronger connectivity, meaning that the genes were more critical. A total of 36 structural genes and 40 TFs were highly co-expressed. Among structural genes, *GAPA* was most related to TFs, followed by *RPIA*, *RBCS_1*, *GADPH*, *HPR*, *AUX1*, *SNRK2_2*, and *CYCD3*, both of which were genes of the carbon metabolism and plant hormone (auxin, abscisic acid, brassinosteroid) signal transduction pathways. In the lignin biosynthesis pathway, *HCT* had higher connectivity with TFs. Forty structural gene-related TFs belonged to 8 TF families, of which the MADS family had the most structural genes linkage, followed by the AP2/ERF and bHLH families.

### Expression pattern analysis of candidate genes during the development of tree peony annual shoots

The expression level of eight randomly selected DEGs in different developmental parts was analysed using qRT-PCR, and the results were basically consistent with the trend of RNA-seq data, indicating that transcriptome data was accurate (Fig. S9).

**Figure 9:**
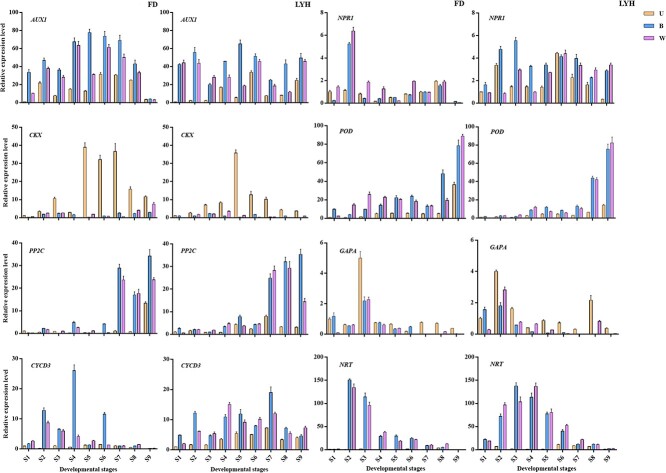
qRT-PCR analysis of genes involved in plant hormone, lignin, carbon, and nitrogen metabolism pathways in tree peony annual shoots at nine developmental stages. FD, *P*. *ostii* ‘Fengdan’; LYH, *P. suffruticosa* ‘Luoyanghong’; U, upper internodes of flowering shoot; B, bottom internodes of flowering shoot; W, whole internodes of vegetative shoot; S1–S9, nine developmental stages. Data are represented as mean ± SD (*n* = 3).

The expression profiles of candidate genes related to phytohormone, lignin, carbon and nitrogen pathways were further examined in tree peony annual shoots. *AUX1*, *CKX*, *PP2C*, *CYCD3*, and *NPR1* were key genes of auxin, cytokinin, abscisic acid, brassinosteroid, and salicylic acid pathways, respectively, which had significant differences in different developmental parts of tree peony annual shoots (Fig. 9). Expression level analysis demonstrated that the expression patterns of *AUX1*, *CKX*, *PP2C*, *CYCD3*, and *NPR1* in the two cultivars were similar. During the development of tree peony annual shoots, the expression levels of *AUX1*, *CYCD3*, and *NPR1* first increased and then decreased, and their expression levels were higher in B and W than in U, which was consistent with the accumulation of IAA. *CKX* was expressed at 40 DAF (S5) to 80 DAF (S7), with the highest expression level at S5 and was mainly expressed at U, while the expression level was lower at B and W, with a difference of more than 150 times. It could degrade cytokinin, which was consistent with the change of cytokinin content in different parts. The expression level of *PP2C* was low before S7 and increased sharply at the late developmental stages of tree peony annual shoots (after S7), and was higher in B and W than that in U, which was consistent with abscisic acid accumulation.

In addition, qRT-PCR analysis was performed on candidate genes of lignin, carbon, and nitrogen metabolism pathways with significant differences in different developmental parts of tree peony annual shoots (Fig. 9). The results demonstrated that the expression level of *POD*, the key gene for lignin biosynthesis, first increased and then decreased before S7, and its expression level was higher in B and W than that in U, which was consistent with the accumulation of lignin. Further, the expression level of *POD* of B and W increased sharply after S7, and the lignin accumulated at this time enhanced its lignification degree, resulting in a different cell fate from that of U. The expression level of *GAPA*, the key gene of carbon metabolism, was highest before and after flowering (S3), and its expression level was higher in U than in B and W. The expression level of *NRT*, the key gene for nitrogen metabolism, was higher in B and W than in U. Except for the first two developmental stages, the expression level showed a gradually decreasing trend, and was highest before and after flowering (S3), which was consistent with the law of carbon and nitrogen transport. This indicated that the carbon source from U was transported to the reproductive organs for flowering and fruiting.

## Discussion

Plants evolve in various unique morphogenesis to adapt different environments, for tree peony, its annual shoot withering characteristics make it unique as a woody shrub. Based on the anatomy, physiology, metabolome, and transcriptome analysis, namely, the development and withering of tree peony annual shoots were co-regulated by a variety of factors, including lignification degree, lignin accumulation, carbon and nitrogen transportation, phytohormone levels, and related various gene expression profiles.

### Regulatory mechanism of the development and withering of tree peony annual shoots

The phenylpropanoid pathway is important for lignin biosynthesis [45]. Compared with young tissues, the mature part of bamboo shoots expressed more genes related to the phenylpropanoid biosynthesis pathway [46], which were the same as the expression pattern of different developmental parts of tree peony annual shoots. Compared with U, cell wall thickening, lignification degree, and lignin accumulation in B and W were stronger. Similarly, the lignification of Moso bamboo culms primarily occurred at the base [47]. The lignin content of tree peony annual shoots increased as the lignification degree increased. Therefore, herb stems with low lignification accumulated less lignin, which could eventually wither due to their inability to withstand the cold.

The carbon content of internodes at the base affects rice stem strength [48, 49], and also affects the synthesis of lignin and cellulose [50, 51]. In addition, a high nitrogen content inhibits the accumulation of carbohydrates and lignin, and more carbon skeletons are used for amino acids synthesis, which are then transported to nitrogen metabolism [52]. In this study, the higher carbon content in B and W promoted the synthesis of lignin and cellulose, resulting in thicker cell walls and higher lignification, reverse pattern was observed in U. Previous studies have demonstrated that the transportation of nutrients from corn stalks to reproductive organs is crucial in the formation of grain yield [53]. In this study, the C/N ratio of B and W was consistently higher than that of U, which may be due to the extremely energy-intensive process during flowering and fruiting of tree peony, while the carbon source of U was transported to the reproductive organs for flowering and fruiting. The same results were also found in *Brassica napus* [54].

### Phytohormone accumulation of tree peony annual shoots

Phytohormones are crucial for the development of tree peony annual shoots. As an important plant hormone, auxin promotes cell elongation, regulates root cap and vascular development, and maintains apical dominance [55]. This study demonstrated that the contents of IAA and IAA-Glu were significantly higher in B and W of tree peony annual shoots than in U, and the contents of ICA and TRP were significantly higher in U than in B and W, while the total auxin content was mainly accumulated in U, which was consistent with the results of auxin distribution in bamboo shoots found by Gamuyao et al [46]. AUX1 encode auxin transporters and play a regulatory role in auxin-dependent plant development [56]. Aux/IAA is an auxin early response gene family, which is involved in the regulation of plant morphogenesis, establishing an auxin concentration gradient for organ and tissue development [57], participating in the auxin response process, and regulating the expression of ARF [58]. As an auxin response factor, ARF can adjust the formation of vascular tissue [59]. *GH3* is the gene of the auxin metabolism process, which maintain auxin homeostasis in plants [60]. SAUR is involved in cell expansion, the early reaction of auxin and its transport [61]. In this study, the expression patterns of *AUX1*, *Aux/IAA*, *ARF*, *GH3*, and *SAUR* in different developmental parts were consistent with the results of IAA accumulation.

Cytokinin can promote cell division, flower bud differentiation, and break apical dominance [62]. The results of this study demonstrated that BAP, tZ, tZOG, and cZ9G were mainly accumulated in B and W, while IPR and oTR were mainly accumulated in U, and the total content of cytokinin was mainly distributed in B and W, which indicated that cytokinin thickened sclerenchyma cells by increasing cell division and cell number, increasing the stem diameter and wall thickness of B and W. This was consistent with the findings in rice that cytokinin signaling was correlated with the thickness of secondary cell wall [63]. CKX inactivated cytokinin by degrading it [64]. In this study, *CKX* was mainly expressed in U, and ARR as the regulator of the cytokinin response pathway, mainly expressed in B and W, indicating that there were more cytokinin in these parts. These results were consistent with the changes of cytokinin content in different developmental parts of annual shoots.

Gibberellin promotes cell wall extension and dormant bud germination [65, 66]. In this study, the contents of GA_1_ and GA_24_ in U were significantly lower than in B and W, while GA_3_ and GA_4_ were the opposite. PIF3 is a phytochrome interacting protein that sense gibberellin signals and mediate photo-regulatory response. It belongs to the bHLH family, and its expression level is negatively correlated with the concentration of GA_3_ [67]. In this study, GA_3_ was mainly accumulated in U, while *PIF3* was mainly expressed in B and W, showing a negative correlation, which was consistent with *Hippophae rhamnoides* subsp. *sinensis* [68].

ABA can regulate seed dormancy, senescence, abscission, and response to stress [69]. During the development of tree peony annual shoots, it was accumulated at B and W. Among the relevant synthetic pathways, PP2C, SNRK2, and ABF promoted the ABA biosynthesis, and all these genes had positive regulatory effects on the formation of lotus rhizome [70].

### Comparative analysis of transcriptome

Tree peony has a large genome and high heterozygosity, which makes it difficult to study. Full-length transcriptome sequencing provides a strategy for the research of such plant, and has been applied to *P. lactiflora* and other plants [71–74]. In this study, the PacBio cDNA library was constructed and 68947 and 66929 full-length transcript sequences were obtained from FD and LYH, respectively, which laid the foundation for identifying key genes responsible for the development of its annual shoots. On this basis, the stem tissues of the two cultivars from three developmental parts were sequenced by RNA-seq, and 36082275 clean reads were obtained on average, accounting for 88.14% of the average number of reads compared with transcripts. In addition, many DEGs were obtained between B/W and U of the two cultivars, as well as many differential metabolites of plant hormones, which fully explained the unique withering characteristics of tree peony annual shoots.

Lignin biosynthesis genes are classified according to their location and function. *PAL*, *C4H*, and *4CL* are located in the upstream of phenylpropanoid biosynthesis pathway, and significantly affect lignin content [75]. Their low expression level in transgenic plants led to the reduction of lignin content [76, 77]. *HCT*, *C3'H*, *F5H*, *COMT*, and *CCoAOMT* affect the biosynthesis of lignin monomers, thus affecting the total lignin content to a certain extent [78]. Inhibition of *COMT* or *F5H* would significantly reduce the content of S-lignin in jute or *Arabidopsis* [79, 80]. *CCR*, *CAD*, and *POD* are located at the downstream of lignin biosynthesis pathway, responsible for the biosynthesis and polymerization of lignin monomers, and are significantly related to lignin content [78, 81]. In this study, FD/LYH were screened as 39/50 DEGs related to lignin biosynthesis based on different developmental parts, including 3/2 *PAL*, 0/1 *C4H*, 0/1 *C3'H*, 4/7 *COMT*, 2/4 *F5H*, 3/6 *4CL*, 0/2 *CCoAOMT*, 2/5 *HCT*, 1/0 *CSE*, 1/0 *CCR*, 12/5 *CAD*, and 11/17 *POD*, respectively. Their expression patterns in different developmental parts of annual shoots were consistent with variation of lignin content. These genes might regulate the lignification and withering process of tree peony annual shoots, providing a theoretical basis for changing the degree of lignification in stems through genetic engineering.

In addition, transcriptional factor family members such as MYB, NAC, and WRKY TFs regulate lignin biosynthesis [82]. Among them, MYB and WRKY families affect lignin deposition by regulating lignin biosynthesis genes *C4H*, *4CL*, and *CCR* [83]. In *Arabidopsis*, MYB4 inhibits the expression of *C4H* and *4CL* [84], while *CCR* is activated by MYB46 [85, 86]. WRKY12 is a transcriptional repressor in *Arabidopsis* that could lead to ectopic lignin deposition [87]. NAC family controls the downstream TFs to perform functions [88]. In this study, 25, 3, and 9 TFs of MYB, NAC, and WRKY families were identified, respectively. However, whether they regulate the development of tree peony annual shoots requires further investigation.

## Conclusions

Tree peony annual shoots accumulated a high amount of lignin and increased numerous xylem cells during their dynamic development. Compared with the upper internodes of flowering shoot, the bottom ones and the whole internodes of vegetative shoot had a higher degree of lignin deposition and showed obvious sclerenchyma. The low content of lignin, cellulose, and C/N ratio in the upper internodes of flowering shoot resulted in the withering of tree peony annual shoots. The co-expression networks of genes for key enzymes and transcription factors related to the growth and development of annual shoots were constructed by analyzing the full-length transcriptomes and comparative transcriptomes at different developmental parts of *P*. *ostii* ‘Fengdan’ and *P. suffruticosa* ‘Luoyanghong’. In addition, the candidate genes were closely related to lignin biosynthesis, carbon and nitrogen metabolism, and plant hormone signaling pathways were screened. This study determined that the dynamic changes of morphology, secondary cell wall composition, C/N ratio, phytohormones, and related gene expression levels varied in different parts of annual shoots during the annual growth cycle. This study revealed the developmental dynamics and withering characteristics of tree peony annual shoots, and provided a theoretical foundation for germplasm innovations and the mechanized harvesting of tree peony annual shoots.

## Materials and methods

### Plant materials

FD and LYH were used as experimental materials, and they grew in Institute of Botany, Chinese Academy of Sciences; there were obvious differences in the withering of their annual shoots. Based on the phenological periods of tree peonies, the development of annual shoots was divided into nine developmental stages [89]. The collected stems were further dissected into upper (U) and bottom (B) internodes according to the absence or presence of axillary buds, respectively. At the same time, the whole (W) stem with apical bud was also collected.

### Phenotypic characteristics determination

Stem length, diameter, and weight were determined with tape, vernier caliper, and balance, respectively. Stem water content and the ratio of actual annual growth of shoots to annual growth were also calculated.

### Anatomic analysis of the developmental process of annual shoots

The fresh stem tissue sliced by hand was treated with phloroglucinol staining solution (1% phloroglucinol (w/v), 12% HCl) for approximately 5 minutes, and the lignified cell tissues were immediately subjected to color reaction and microscopic observation.

Stem samples were collected at nine developmental stages. 5 mm of internodes were fixed in formalin acetic acid, and vacuumed for 20 minutes to completely immerse the samples in the fixative. Next, the samples were dehydrated in gradient ethanol and embedded in resin. One-micrometer sections were obtained by Leica EM UC7 microtome, then stained with toluidine blue, and observed using microscope.

### Secondary cell walls observations

The samples after ethanol dehydration were dried with an automatic critical point dryer, and then glued on the metal platform to observe the desired side. The metal platform was sprayed with gold and coated with ion sputtering instrument. Secondary cell walls were observed by field emission scanning electron microscopy. The cell wall thickness of xylem cells was measured by ImageJ software.

### Lignin, cellulose, hemicellulose, carbon, and nitrogen content determinations

The contents of lignin, cellulose, and hemicellulose were determined as previously described with some modifications [90, 91]. Acetyl bromide was used to dissolve the lignin in annual shoots, and the total lignin content was determined by colorimetry. The cell wall material was hydrolysed by trifluoroacetic acid, and the unhydrolysed and hydrolysed substances were determined with the anthrone method. The sugar content obtained was cell wall cellulose content and total hemicellulose content, respectively.

The carbon and nitrogen contents of stem samples were determined by elemental analyser Vario EL III.

### Detection of phytohormones

The content of phytohormones (auxin, gibberellin, cytokinin, abscisic acid, ethylene, and strigolactone) in the three developmental parts of the annual shoots of FD and LYH was determined by MetWare (http://www.metware.cn/). Significantly regulated metabolites were determined by absolute Log_2_FC (fold change). Then, KEGG enrichment analysis of differential metabolite was carried out.

### PacBio Iso-Seq library preparation, sequencing, and data analysis

The mixed plant tissues of FD and LYH during the annual growth season were used for full-length transcriptome analysis. Total RNA was extracted with TRIzol reagent (Omega BioTek, GA, USA). After qualified samples were detected, full-length cDNA was synthesized, followed by PCR amplification, terminal repair, splicing, exonuclease digestion, and a sequencing library was obtained. After the library was validated, full-length transcriptome sequencing was performed using PacBio RS II platform. After a series of processing, the full-length transcripts were functionally annotated [92].

### Illumina transcriptome library preparation, sequencing, and data analysis

Eighteen cDNA libraries were constructed from three developmental parts of FD and LYH stem tissues. The cDNA library was obtained by terminal repair, poly-A tail addition, PCR enrichment, and splicing. After validating the library, Illumina Hiseq X-Ten platform was used for transcriptome sequencing. Based on PacBio reference genome, clean reads were further analysed [93]. The gene expression level was estimated by fragments per kilobase of transcript per million fragments mapped (FPKM) [94]. Differential expression analysis was performed using DESeq2 [95]. Genes with false discovery rate (FDR) < 0.01 and FC ≥ 2 or FC ≤ 0.5 were assigned as differentially expressed.

### Gene expression analysis

Total RNA was extracted with TRIzol reagent. cDNA template was prepared with HiScript II reverse transcriptase kit (Vazyme, Nanjing, China). Quantitative real-time PCR (qRT-PCR) was used to analyse gene expression levels through StepOne Real-Time PCR System (Applied Biosystems, Carlsbad, USA), and 2 × M5 HiPer Realtime PCR Super mix (Mei5bio, Beijing, China) was used in qRT-PCR reactions. Relative quantification was determined using the 2^−ΔΔCt^ method [96]. The primers used are listed in Table S4.

### Statistical analysis

One-way analysis of variance was used to assess differences using SPSS 24.0. Differences were considered significant at *p* < 0.05. Graphs were drawn with GraphPad Prism 7.00. All experiments included at least three biological replicates.

## Acknowledgements

We thank Mr. Zhuxin Wei, Luoyang Zongheng Horticulture Co., LTD., for his help in collecting tree peony materials. We are also grateful to Mrs. Fengqin Dong and Xiuping Xu from Plant Science Facility of Institute of Botany, Chinese Academy of Sciences for providing semi-thin sectioning and scanning electron microscopy technical assistance, respectively. This work was supported by the Strategic Priority Research Program of Chinese Academy of Sciences (grant XDA23080601).

## Author contributions

Z.L., B.W. and N.T. designed the experiments. N.T. conducted the experiments and wrote the manuscript. N.T. and L.P. performed data analyses. L.P. and Q.S. revised the manuscript. All the authors read and approved the final manuscript.

## Data availability

The data underlying this article are available in the article and in its online supplementary material.

## Conflict of interest statement

The authors declare that they have no conflict of interest.

## Supplementary Data

Supplementary data is available at *Horticulture Research* online.

## Supplementary Material

Web_Material_uhad152
