# Supplementary material for: Histology, physiology, and transcriptomic and metabolomic profiling reveal the developmental dynamics of annual shoots in tree peonies (Paeonia suffruticosa Andr.)
Source: Hortic Res. 2023 Aug 1;10(9):uhad152. doi: 10.1093/hr/uhad152 (PMC10493643; doi:10.1093/hr/uhad152)
Supplement: Web_Material_uhad152 [file web_material_uhad152.docx]

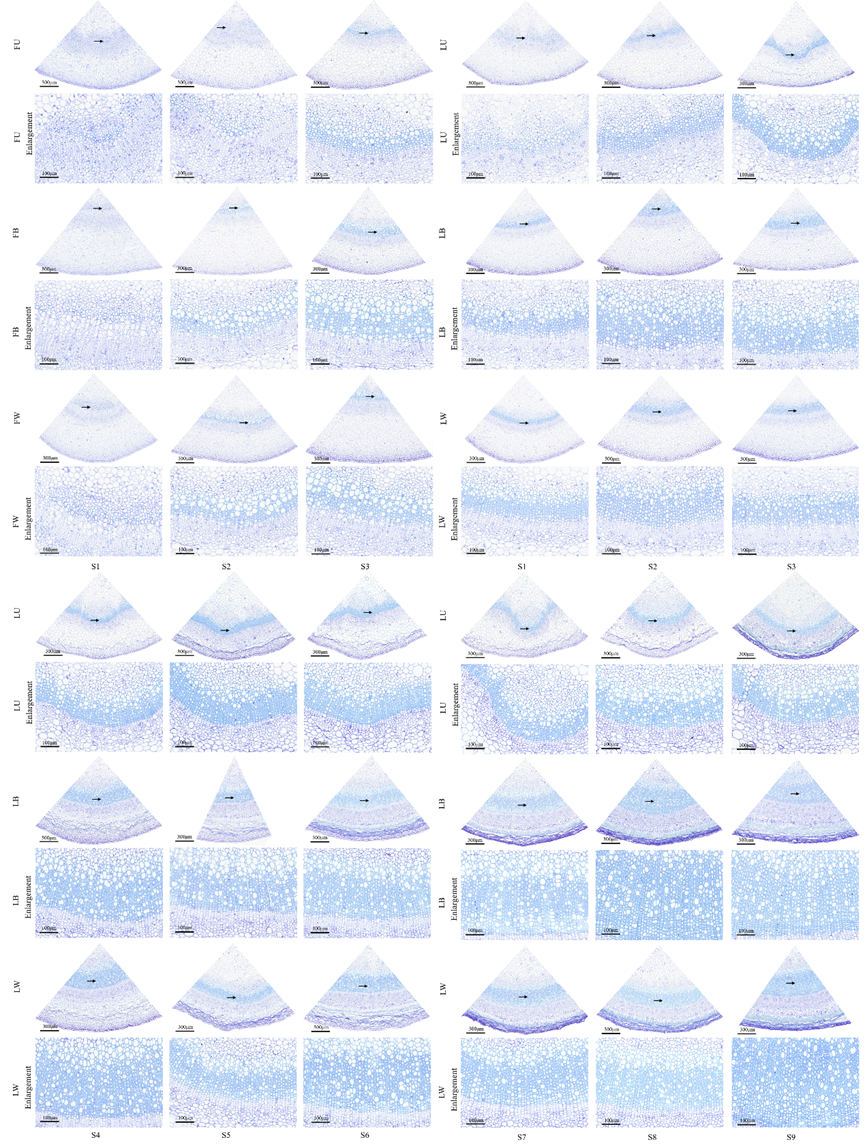


**Fig. S1 Anatomic analysis of tree peony annual shoots.** The micrographs of partial enlargement of regions are marked by black arrows. FU/FB/FW/LU/LB/LW, bars = 300 μm; Enlargement, bars = 100 μm. FU/FB/FW, upper internodes of flowering shoot/bottom internodes of flowering shoot/whole internodes of vegetative shoot in *P. ostii* ‘Fengdan’; LU/LB/LW, upper internodes of flowering shoot/bottom internodes of flowering shoot/whole internodes of vegetative shoot in *P. suffruticosa* ‘Luoyanghong’; S1, extractive branch stage; S2, unfolding leaves stage; S3, flowering stage; S4, 20 days after flowering (DAF); S5, 40 DAF; S6, 60 DAF; S7, 80 DAF; S8, maturity stage; S9, withering stage.


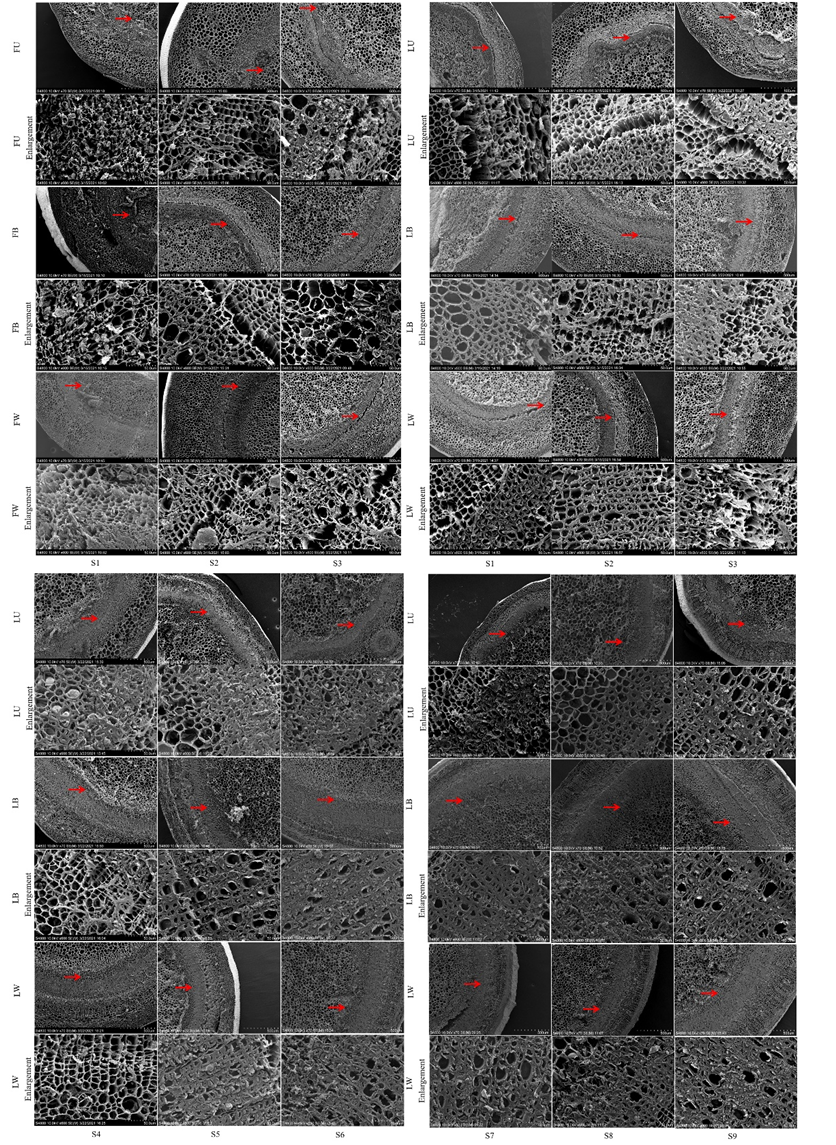


**Fig. S2 SEM** [**observation**](javascript:;) **of tree peony annual shoots.** The micrographs of partial enlargement of regions are marked by red arrows. FU/FB/FW/LU/LB/LW, bars = 500 μm; Enlargement, bars = 50 μm. FU/FB/FW, upper internodes of flowering shoot/bottom internodes of flowering shoot/whole internodes of vegetative shoot in *P. ostii* ‘Fengdan’; LU/LB/LW, upper internodes of flowering shoot/bottom internodes of flowering shoot/whole internodes of vegetative shoot in *P. suffruticosa* ‘Luoyanghong’; S1, extractive branch stage; S2, unfolding leaves stage; S3, flowering stage; S4, 20 days after flowering (DAF); S5, 40 DAF; S6, 60 DAF; S7, 80 DAF; S8, maturity stage; S9, withering stage.


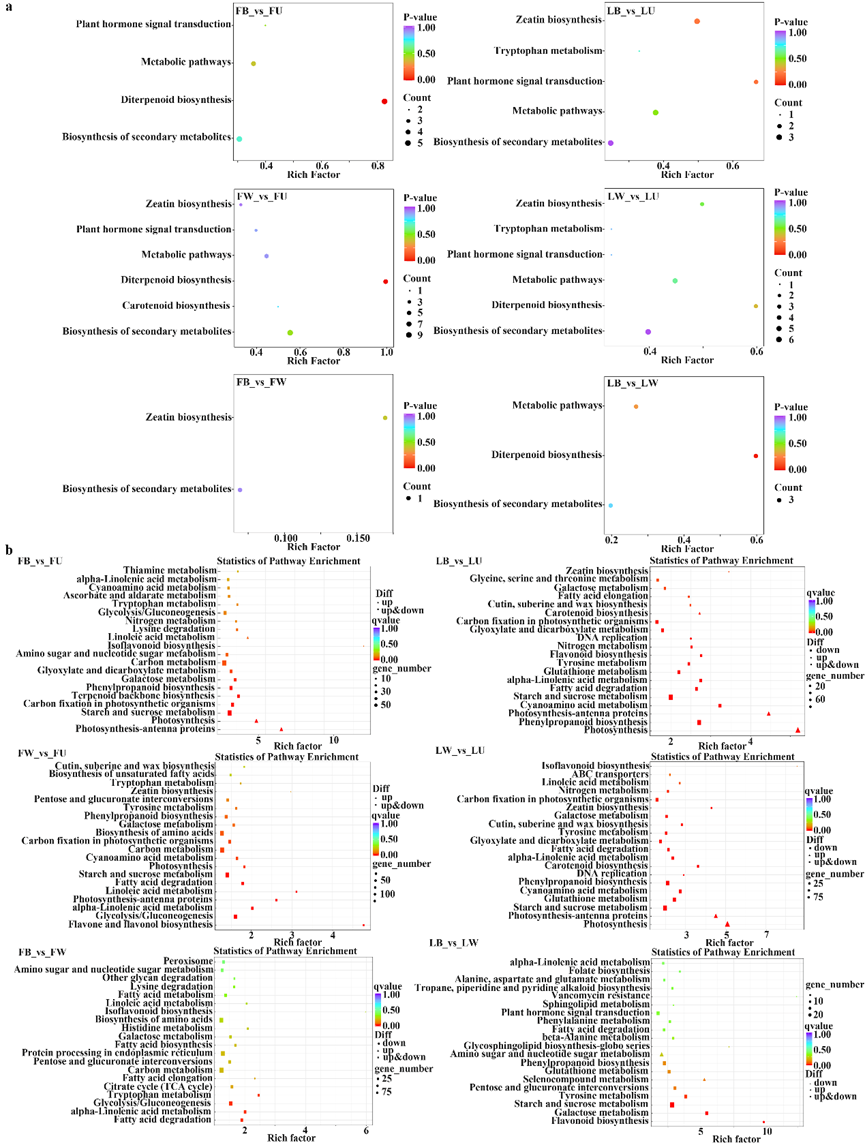


**Fig. S3** **KEGG enrichment diagrams of differentially accumulated metabolites and DEGs of each comparison group.** **a** KEGG enrichment diagrams of differentially accumulated metabolites. **b** KEGG enrichment diagrams of DEGs. FU/FB/FW, upper internodes of flowering shoot/bottom internodes of flowering shoot/whole internodes of vegetative shoot in *P. ostii* ‘Fengdan’; LU/LB/LW, upper internodes of flowering shoot/bottom internodes of flowering shoot/whole internodes of vegetative shoot in *P. suffruticosa* ‘Luoyanghong’.


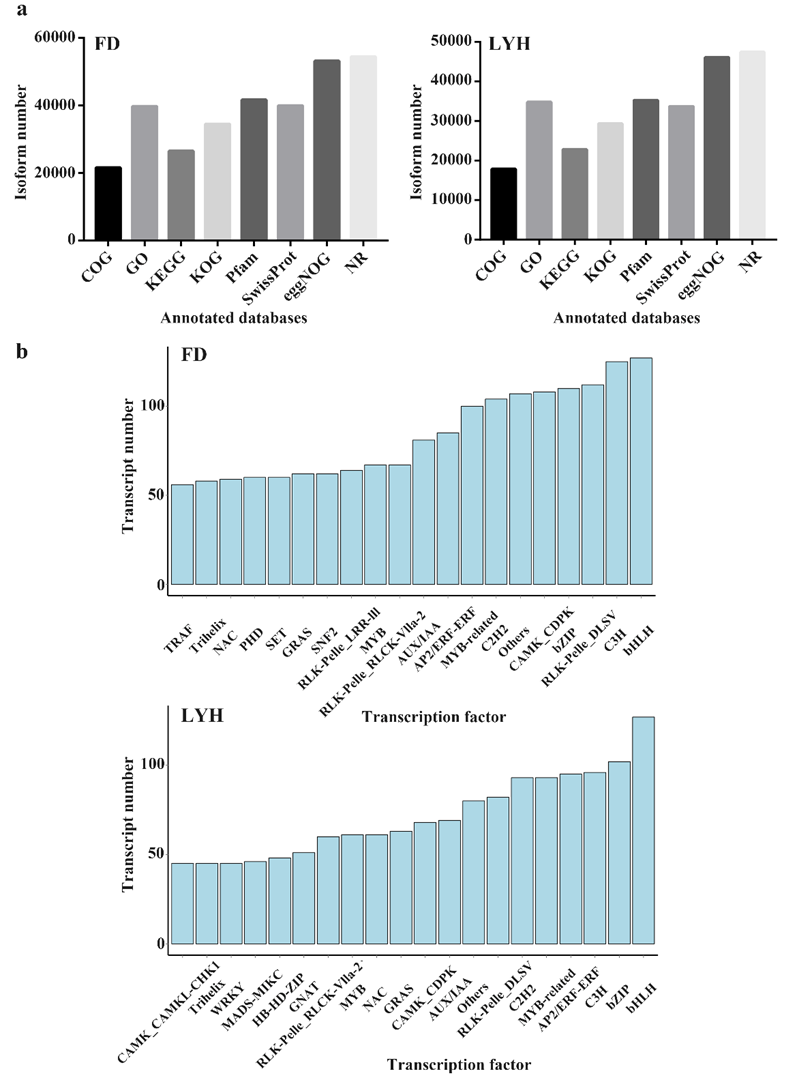


**Fig. S4 Gene annotation and TFs prediction of full-length isoforms.** **a** Histogram of full-length isoforms annotated in the COG, GO, KEGG, KOG, Pfam, SwissProt, eggNOG, and NR public databases. **b** Statistics on the number of transcription factor families predicted by full-length isoforms. FD, *P. ostii* ‘Fengdan’; LYH, *P. suffruticosa* ‘Luoyanghong’.


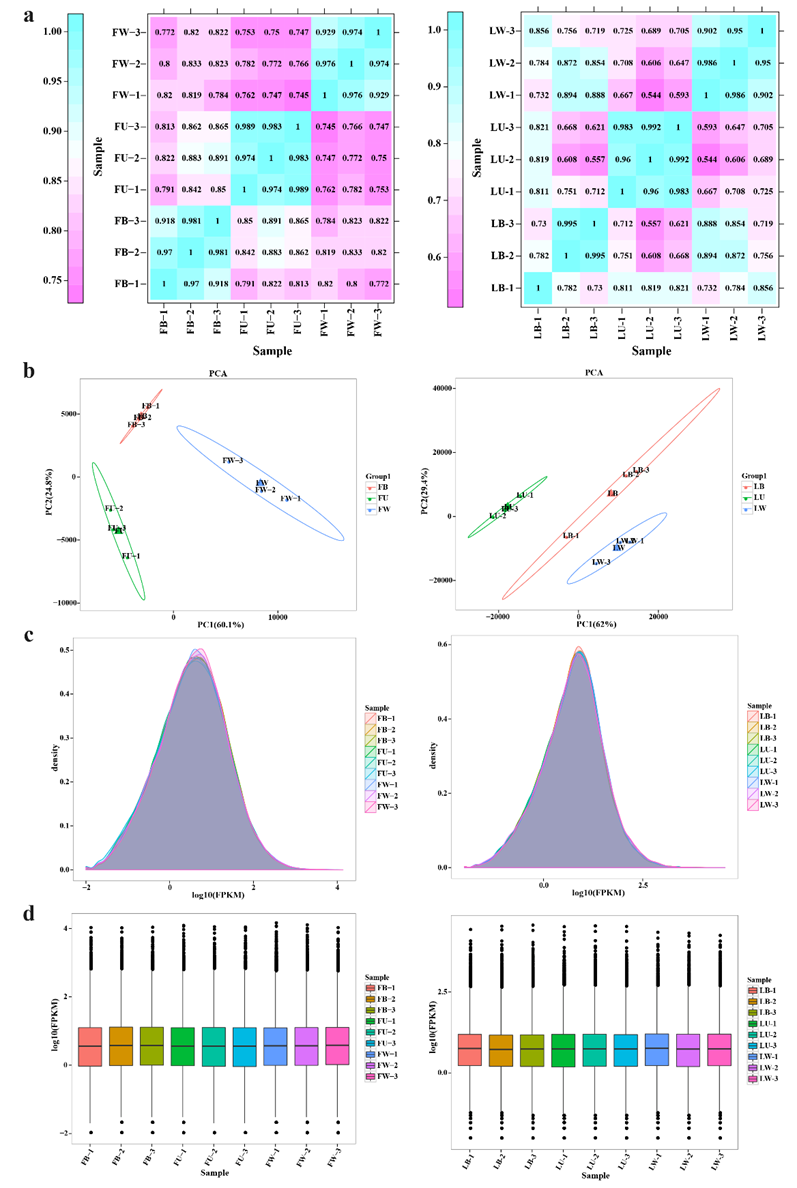


**Fig. S5 Global gene expression analysis.** **a** Pearson correlation between samples based on expression levels. **b** PCA between samples based on expression levels. **c** Density plot displaying the gene density at different FPKM levels. **d** Box plot of the FPKM distribution among samples. FU/FB/FW, upper internodes of flowering shoot/bottom internodes of flowering shoot/whole internodes of vegetative shoot in *P. ostii* ‘Fengdan’; LU/LB/LW, upper internodes of flowering shoot/bottom internodes of flowering shoot/whole internodes of vegetative shoot in *P. suffruticosa* ‘Luoyanghong’.


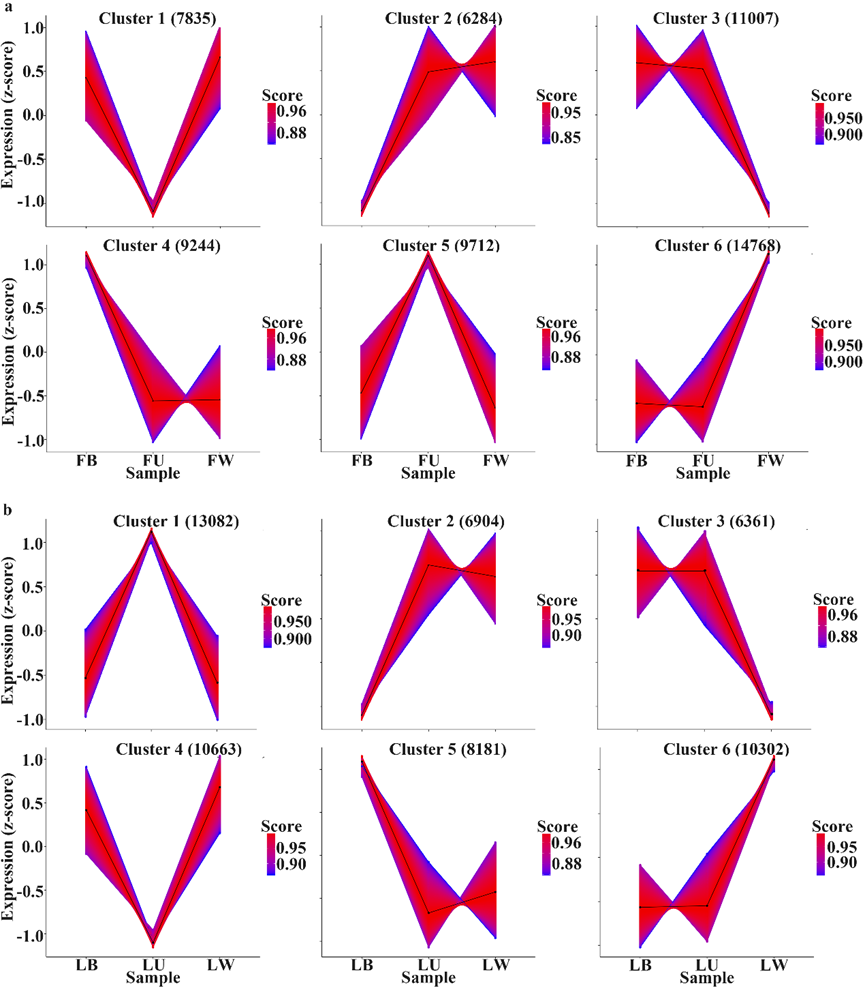


**Fig. S6 K-means cluster diagrams.** **a** Six clusters were identified based on expression levels in three developmental parts (FB, FU and FW) of *P. ostii* ‘Fengdan’. **b** Six clusters were identified based on expression levels in three developmental parts (LB, LU and LW) of *P. suffruticosa* ‘Luoyanghong’. FU/FB/FW, upper internodes of flowering shoot/bottom internodes of flowering shoot/whole internodes of vegetative shoot in *P. ostii* ‘Fengdan’; LU/LB/LW, upper internodes of flowering shoot/bottom internodes of flowering shoot/whole internodes of vegetative shoot in *P. suffruticosa* ‘Luoyanghong’.


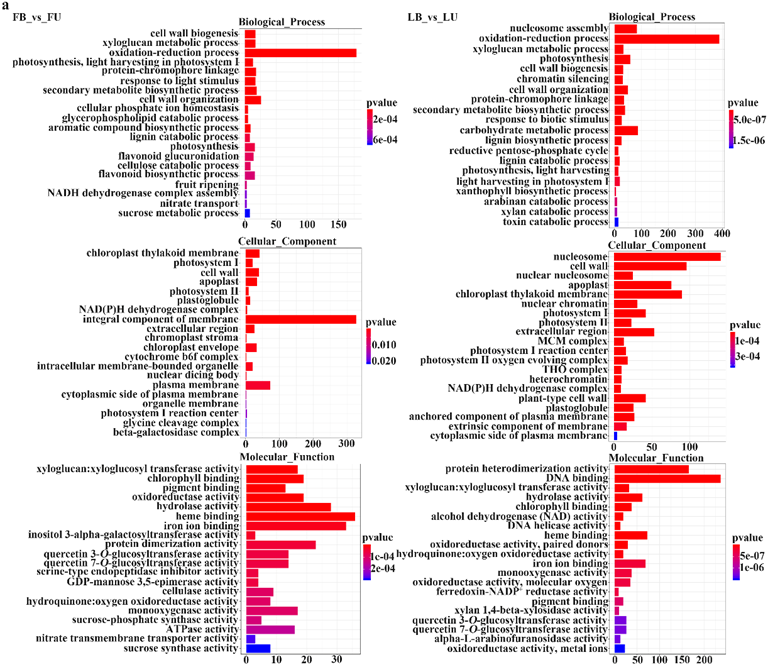


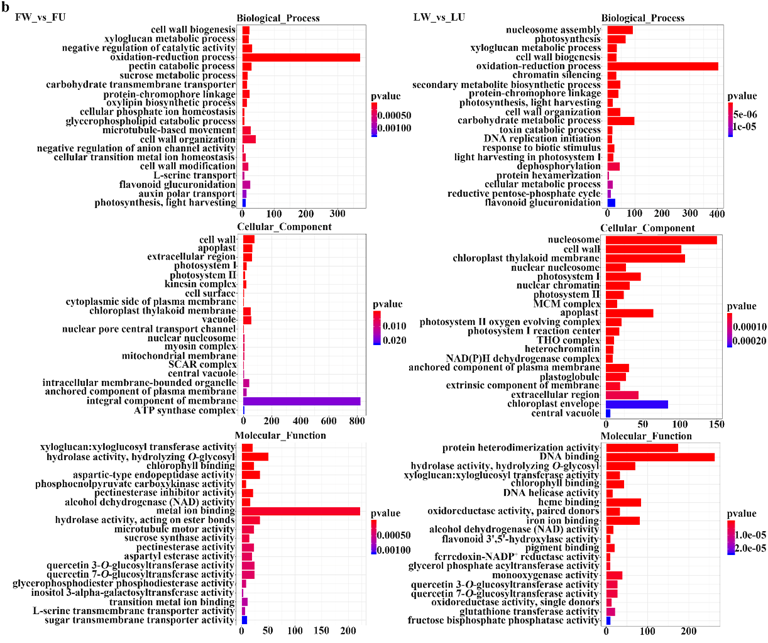


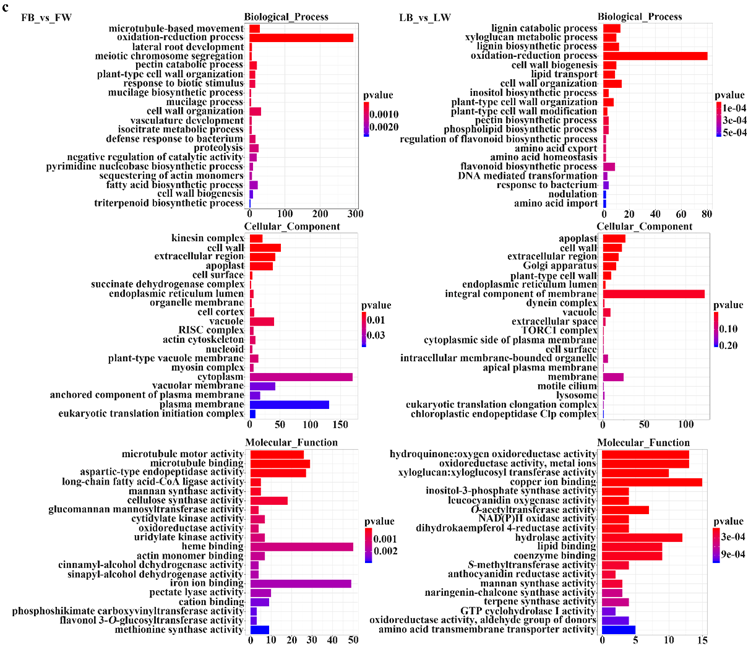


**Fig. S7 GO enrichment analysis of DEGs.** **a** FB_vs_FU and LB_vs_LU. **b** FW_vs_FU and LW_vs_LU. **c** FB_vs_FW and LB_vs_LW. FU/FB/FW, upper internodes of flowering shoot/bottom internodes of flowering shoot/whole internodes of vegetative shoot in *P. ostii* ‘Fengdan’; LU/LB/LW, upper internodes of flowering shoot/bottom internodes of flowering shoot/whole internodes of vegetative shoot in *P. suffruticosa* ‘Luoyanghong’.


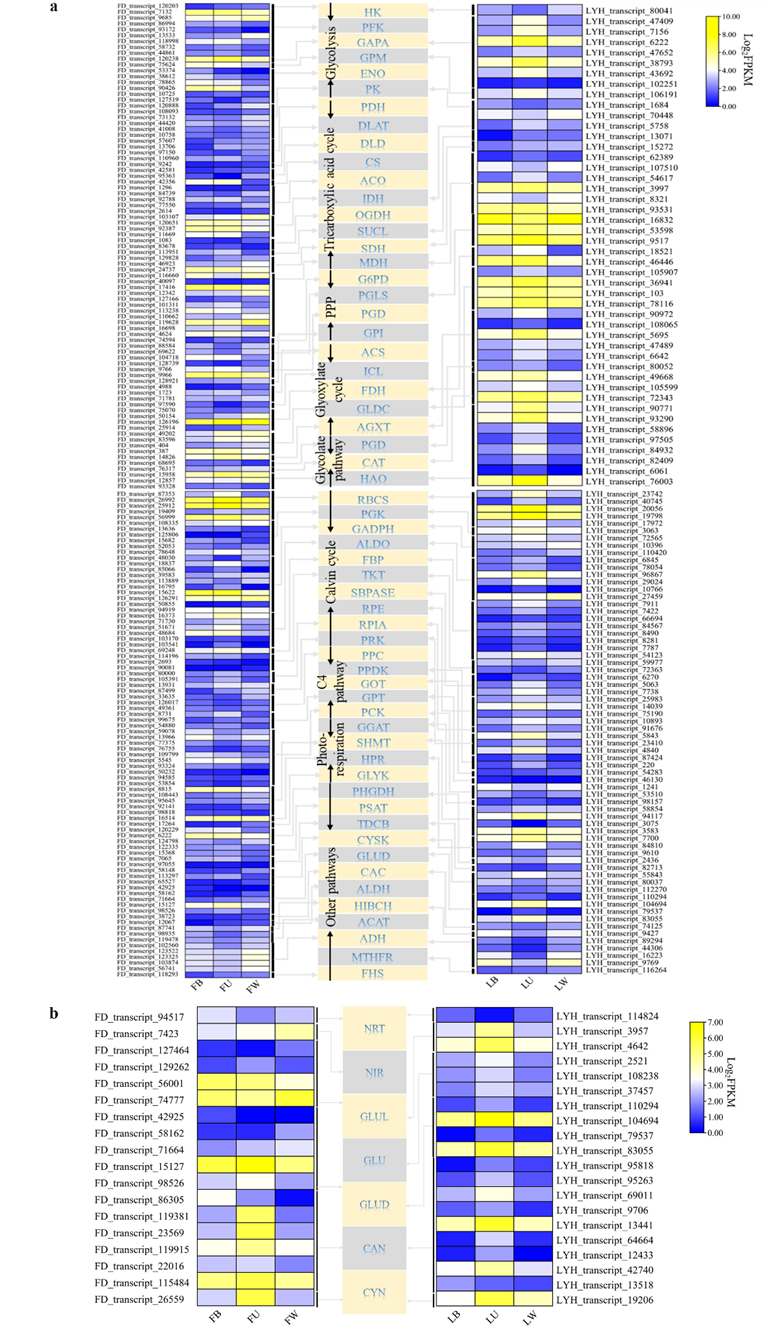


**Fig. S8 Identification and analysis of DEGs in the three developmental parts of annual shoots of two tree peony cultivars.** **a** Expression analysis of DEGs related to carbon metabolism pathway. **b** Expression analysis of DEGs related to nitrogen metabolism pathway. FU/FB/FW, upper internodes of flowering shoot/bottom internodes of flowering shoot/whole internodes of vegetative shoot in *P. ostii* ‘Fengdan’; LU/LB/LW, upper internodes of flowering shoot/bottom internodes of flowering shoot/whole internodes of vegetative shoot in *P. suffruticosa* ‘Luoyanghong’.


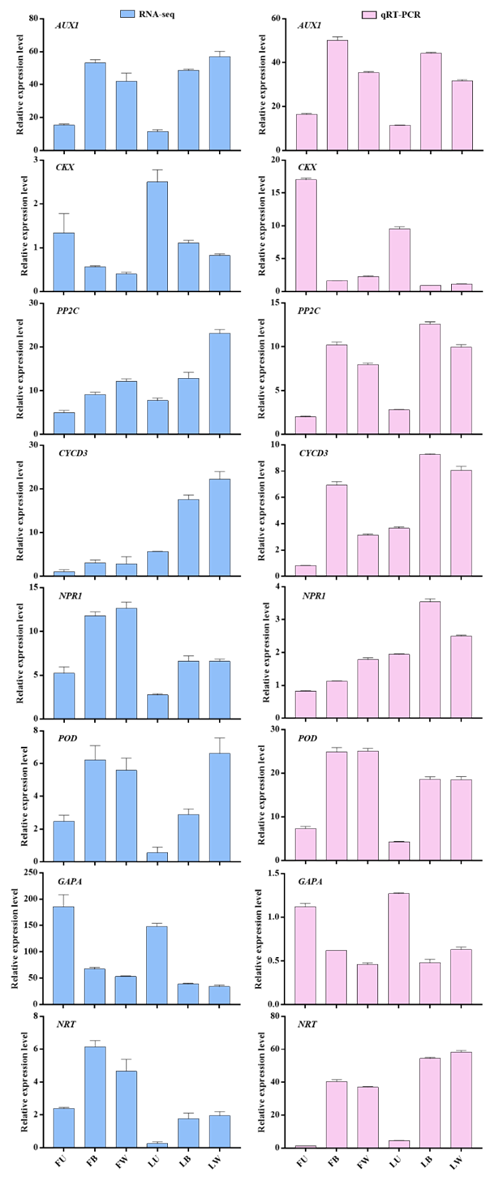


**Fig. S9 Gene relative expression levels of RNA-seq and qRT-PCR.** FU/FB/FW, upper internodes of flowering shoot/bottom internodes of flowering shoot/whole internodes of vegetative shoot in *P. ostii* ‘Fengdan’; LU/LB/LW, upper internodes of flowering shoot/bottom internodes of flowering shoot/whole internodes of vegetative shoot in *P. suffruticosa* ‘Luoyanghong’.

**Table S1. Statistics of the full-length transcriptome data.**

| Samples | CCS Number | Number of full-length non-chimeric reads | Number of consensus isoforms | Number of polished high-quality isoforms | Full-  length isoforms |
| --- | --- | --- | --- | --- | --- |
| FD | 375,511 | 320,140 | 130,166 | 130,159 | 68,947 |
| LYH | 405,384 | 297,959 | 116,361 | 116,354 | 66,929 |

**Table S2. Alignment results between RNA-seq data and the full-length transcriptome data.**

| Sample | Total clean reads | Total mapping (%) | Uniquely mapping (%) |
| --- | --- | --- | --- |
| FB-1 | 37,266,156 | 88.25 | 38.33 |
| FB-2 | 31,380,464 | 87.19 | 38.27 |
| FB-3 | 32,770,573 | 87.70 | 38.83 |
| FU-1 | 36,669,929 | 89.29 | 39.07 |
| FU-2 | 38,110,195 | 88.05 | 38.64 |
| FU-3 | 43,841,236 | 87.70 | 38.44 |
| FW-1 | 31,948,578 | 89.08 | 39.06 |
| FW-2 | 34,170,476 | 89.41 | 39.65 |
| FW-3 | 32,172,720 | 89.42 | 38.92 |
| LB-1 | 34,599,166 | 87.50 | 49.22 |
| LB-2 | 35,264,607 | 87.47 | 48.98 |
| LB-3 | 36,307,855 | 86.66 | 48.85 |
| LU-1 | 39,288,558 | 87.99 | 50.69 |
| LU-2 | 35,654,291 | 87.44 | 50.49 |
| LU-3 | 41,510,021 | 87.88 | 50.35 |
| LW-1 | 35,589,819 | 87.48 | 50.84 |
| LW-2 | 41,009,420 | 89.45 | 51.99 |
| LW-3 | 31,926,890 | 88.58 | 51.62 |
| Average | 36,082,275 | 88.14 | 44.57 |

**Table S3. Statistics on the number of DEGs.**

| DEG Set | DEGs Number | up-regulated | down-regulated |
| --- | --- | --- | --- |
| FB_vs_FU | 1,732 | 937 | 795 |
| FB_vs_FW | 3,945 | 1,931 | 2,014 |
| FW_vs_FU | 4,942 | 2,606 | 2,336 |
| LB_vs_LU | 3,713 | 2,046 | 1,667 |
| LB_vs_LW | 748 | 475 | 273 |
| LW_vs_LU | 4,306 | 2,244 | 2,062 |

**Table S4. The related information of the primers.**

| Gene ID | Primers Name | Primers Pairs (5ʹ→3ʹ) |
| --- | --- | --- |
| FD_transcript_9960 | qAUX1f | ACCGTTGGATCACTTCTCGT |
|  | qAUX1r | CACCGAATCCAAAGCCAACC |
| FD_transcript_100007 | qCKXf | TGGCTCAACCTTCTCATTCCC |
|  | qCKXr | GTTGTCAGTTCCCGAGGAAGA |
| FD_transcript_97457 | qPP2Cf | AGATCACAAGCCGGATCGAC |
|  | qPP2Cr | CCCATAGTCCATCACTCGCC |
| FD_transcript_99390 | qCYCD3f | CACAAGGAGGCTTGGGTTGA |
|  | qCYCD3r | CACAGCCGATAACATGCAGC |
| FD_transcript_122567 | qNPR1f | GCCGAAATGGTGTCTCCTGA |
|  | qNPR1r | GGAGCATTACCACCACTCCC |
| FD_transcript_51026 | qPODf | CTGCCCAAATGCAGAGCCTA |
|  | qPODr | GCTTTGGCGCTGTCTATGATG |
| FD_transcript_120238 | qGAPAf | TACGACTCTACCCTCGGCAT |
|  | qGAPAr | GATGCTTCCCAGCACCATCT |
| FD_transcript_94517 | qNRTf | GCACCGATTGTCTTGTCTGC |
|  | qNRTr | CACCAACGTTAGCCCATCCA |
| EF608942 | qTUBf  qTUBr | TGAGCACCAAAGAAGTGGACGAAC  CACACGCCTGAACATCTCCTGAA |
